# The pathophysiology of “happy” hypoglycemia

**DOI:** 10.1186/s12245-021-00348-7

**Published:** 2021-04-21

**Authors:** Thomas Loeb, Anna Ozguler, Geraldine Baer, Michel Baer

**Affiliations:** 1 SAMU des Hauts-de-Seine, Assistance Publique – Hôpitaux de Paris, Raymond Poincaré Hospital, University of Paris-Saclay, 104 boulevard Raymond Poincaré, 92 380 Garches, France; 2 Department of Emergency Medicine, Corporal Michael J. Crescenz VA Medical Center, University of Pennsylvania Medical Center, Philadelphia, PA USA

**Keywords:** Hypoglycemia, Lactate, Asymptomatic, Emergency medical service

## Abstract

**Background:**

Hypoglycemia usually includes various neurological symptoms, which are the consequence of neuroglycopenia. When it is severe, it is associated with altered mental status, even coma.

**Case presentation:**

We report the case of a patient with severe hypoglycemia, completely asymptomatic, due to the increase of lactate production in response to tissue hypoperfusion following a hemorrhagic shock. This illustrates that lactate can substitute glucose as an energy substrate for the brain. It is also a reminder that this metabolite, despite its bad reputation maintained by its role as a marker of severity in critical care patients, has a fundamental role in our metabolism.

**Conclusions:**

Following the example of the “happy hypoxemia” recently reported in the literature describing asymptomatic hypoxemia in COVID-19 patients, we describe a case of “happy hypoglycemia.”

## Background

Hypoglycemia usually includes various neurological symptoms, which are the consequence of neuroglycopenia. When it is severe, it is associated with altered mental status, even coma.

We report the case of a patient who presented with hemorrhagic shock and severe hypoglycemia who remained completely asymptomatic. This case is an opportunity to explain the physiopathology of this unusual clinical situation and a reminder that glucose is not the only energy substrate for the brain.

## Case presentation

An 85-year-old woman called an emergency medical service (EMS) for hematochezia. Her medical history included atrial fibrillation, hypertension, chronic constipation, and chronic low back pain. Her medications were apixaban 5 mg/day in 2 doses, furosemide 40 mg/day, bisoprolol 10 mg/day, and perindopril 10 mg/day. Her height was 150 cm and weight 42 kg (body mass index 18.7 kg/m^2^). She lived at home with her husband and was completely independent with acts of daily life.

A basic life support ambulance was dispatched. When they arrived, the ambulance crew noted cyanosis in bilateral hands and feet. The patient was awake, alert, and oriented to place, person, and date, and conversant, with a Glasgow Coma Scale of 15. She had been complaining of tiredness, anorexia, and constipation for 8 days and blood in her stools. Blood pressure was 130/40 mmHg, heart rate 143 beats/min, respiratory rate 14 cycles/min, and temperature 37 °C. Oxygen saturation (SpO_2_) was hard to measure with a high variability of the results displayed on the oxymeter. A medical team (advance life support ambulance with an emergency physician on board) was sent for additional support.

When the medical team arrived on the scene, the medical history found that hematochezia had started within the last 24 h, but was also associated, in the past several days, with melena and diffuse abdominal pain. The clinical examination showed signs of tissue hypoperfusion with skin mottling of the lower limbs, peripheral vasoconstriction, increased capillary refill time, and peripheral cyanosis. The skin was very pale. Blood pressure was 112/40 mmHg, heart rate 86 beats/min, respiratory rate 20 cycles/min, temperature 37 °C, and SpO_2_ remained difficult to measure due to severe peripheral vasoconstriction. Placed at the ear, the oxymeter was able to show an SpO_2_ of 90% while the patient was on oxygen at 9 L/min via mask. The electrocardiogram showed atrial fibrillation with inverted T waves in V4, V5, and V6. The hemoglobin, measured twice on a capillary and venous point of care sample, was 5 g/dL. Capillary blood glucose, controlled three times, was not measurable despite the normal level of consciousness of the patient.

Management consisted of oxygen at 9 L/min via high flow mask, intravenous catheterization and rapid intravenous fluid infusion with 500 mL of isotonic saline solution (0.9% NaCl), and intravenous administration of 12 g of glucose with a 30% glucose solution. Anemia correction was deferred. Thirty-nine minutes after the medical team arrival, the patient was transported to an intensive care unit (ICU).

At the hospital, the symptomatology was unchanged. Laboratory results were are follows (normal values in parentheses): pH 7.30 (7.3–7.42), pCO_2_ 30 mmHg (35–45), pO_2_ 297 mmHg (> 90), bicarbonate 15 mmol/L (22–26), sodium 140 mmol/L (136–145), potassium 5.0 mmol/L (3.7–5.2), chloride 108 mmol/L (95–105), glucose 5.9 mmol/L (3.8–6.1), lactate 0.56 mmol/L (0.60–2.00), creatinine 251 μmol/L (55–100), and urea 45.1 mmol/L (2.5–7.5). Hematology was as follows: leukocytes 19,000/mL (4000–10,000), red blood cells 1,980,000/mL (4,000,000–5,2000,000), hemoglobin 4.8 g/dL (12.5–15.5), and platelets 324,000/mL (150,000–400,000). The prothrombin level was 39% (70–125). The apixaban anti-Xa was 484 ng/mL.

Therapies consisted of 50 IU/kg of 4-factor prothrombin complex concentrate to reverse apixaban, transfusion of 6 units of packed red blood cells, and 3 units of plasma. An esogastroduodenal fibroscopy did not show any lesions. A colonoscopy visualized, in the left angle and in the sigmoid colon, an ischemic colitis with ulcerations responsible for the bleeding in the previous days. No active bleeding was seen. The final diagnosis was hemorrhagic shock secondary to intestinal bleeding on ischemic colitis in the context of apixaban overdose, itself complicated by acute kidney injury and metabolic acidosis.

During the remainder of the patient’s stay in the ICU, there was no hemorrhagic or hypoglycemia recurrence, the acid-base balance was restored, and the kidneys recovered normal function. The patient was discharged to the internal medicine department on day 10. Her mental status was closely monitored and remained normal throughout her hospital stay.

## Discussion

Glucose is the main substrate utilized by the brain and many regulatory mechanisms can be activated to maintain an effective glucose concentration. To this end, glucoprivation triggers a complex neuroendocrine response with its fail-safes. In particular, it stimulates many hormone secretions such as corticosteroid releasing hormone, glucagon, or epinephrine. Epinephrine secretion is associated with an increase in lactate concentration related to their direct effects on carbohydrate metabolism. Indeed, elevated blood lactate is viewed as evidence of tissue hypoxia, with lactate levels being proportional to the defect in oxidative metabolism. However, many tissues generate pyruvate and lactate under aerobic conditions (so-called aerobic glycolysis).

When these mechanisms are overwhelmed or fail, hypoglycemia occurs with a range of non-specific symptoms related to dysautonomy or neuroglycopenia [1]. Moreover, symptomatic hypoglycemia is diagnosed using Whipple’s Triad, which combines neuroglycopenic symptoms (such as muscle weakness, sleepiness, dysarthria, confusion, loss of consciousness or seizures), low plasma glucose concentration, and resolution of those symptoms when plasma glucose concentration is raised. With the exception of patients with “hypoglycemia unawareness” who do not perceive the symptoms, the neurological manifestations usually occur at plasma glucose concentrations lower than 3.9 mmol/L [2]. In the most severe cases, hypoglycemia is associated with loss of consciousness or coma. In the absence of rapid correction, hypoglycemia can be dramatic [3] and sometimes leads to death [4]. Hypoglycemia is usually considered severe when plasma glucose concentration is lower than 2.2 mmol/L.

In this case, the patient’s capillary blood glucose was measured in the pre-hospital setting using the Abbott FreeStyle Precision Neo blood glucometer. The glucose concentration reading limits of this device are in the range of 1.1 mmol/L to 27.8 mmol/L. When the result is outside of the reading limits, the display shows “Lo” (for Low) when it is below 1.1 mmol/L and “Hi” (for High) when it is above 27.8 mmol/L. The device used had been checked and calibrated according to the service control procedures, based on the manufacturer’s recommendations. On capillary blood samples taken from three different sites (right and left index fingers and ear), the results displayed were “Lo.” Therefore, the patient had an extremely deep hypoglycemia with a plasma glucose concentration below 1.1 mmol/L and would have typically been comatose [5]. However, she was perfectly awake, alert, and presented no clinical signs, even subtle, of neuroglycopenia.

Glucose has long been considered the only energy substrate for the brain [6]. Numerous works have provided arguments against this hypothesis and suggested, in particular, that lactate was a possible substrate for neurons in a situation of “energy crisis” [7]. Lactate takes over as energy supply of the brain in lack of glucose by intervening in the metabolic coupling between astrocytes and neurons described by Pellerin and Magistretti [8]. It is believed to help maintain synaptic transmission, especially during periods of intense activity. During these periods, astrocytes release large amounts of lactate, produced via glutamate, which is then transferred to the neurons.

Evidence that lactate was interchangeable with glucose to support oxidative metabolism in cortical neurons was both experimentally provided by biochemists more than 20 years ago [9] but also clinically, notably by oncohematologists [10]. Some solid cancers or hematological malignancies lead to an acceleration of the transformation of glucose into lactate within cancer cells. This glucose metabolism deregulation is then accompanied by a rare complication, known as hyperwarburgism, which combines metabolic acidosis due to lactate accumulation and severe hypoglycemia that remains completely asymptomatic.

In this case, the patient had severe anemia and signs of tissue hypoperfusion, two well-known causes of hyperlactatemia [11]. Indeed, in cases of tissue hypoperfusion and cellular hypoxia, as in this patient, the reduction of pyruvate to lactate by the action of lactate dehydrogenase (LDH) is accelerated and leads to hyperlactatemia [12]. According to these references, one would expect the plasma level to be elevated. However, this patient’s lactate was found to be low at 0.56 mmol/L.

And, despite a very likely activation of the neuroendocrine response with its epinephrine secretion, which could explain the patient’s ability to maintain a systolic (but not diastolic) blood pressure, there was a failure of these complex neuroendocrine mechanisms to correct the hypoglycemia. Lactate, which can cross the blood brain barrier, became the substrate of choice for the brain. We therefore hypothesize that the lactate produced under the effect of cellular hypoxia was totally consumed by the brain and served as an energetic substrate to replace glucose and maintain a perfect state of consciousness despite the deep hypoglycemia.

It is, to our knowledge, the first time proposed in the literature of the importance of lactate in brain metabolism in hypoglycemic patients. This case illustrates that, outside the context of hyperwarburgism in oncohematology, lactate can substitute glucose as an energy substrate for the brain. It is also a reminder that this metabolite, despite its bad reputation maintained by its role as a marker of severity in critical care patients [13, 14], has a fundamental role in our metabolism. In the case we are reporting, its overproduction avoided the serious manifestations of neuroglycopenia. Thus, following the example of the “happy hypoxemia” recently reported in the literature describing asymptomatic hypoxemia in COVID-19 patients, we describe a case of “happy hypoglycemia” [15].

## Data Availability

All data generated or analyzed during this study are included in this published article.

## Abbreviations

0.9% NaCl: Isotonic saline solution
COVID-19: Coronavirus disease 2019
ICU: Intensive care unit
EMS: Emergency medical service
LDH: Lactate dehydrogenase
pCO_2_: Partial pressure of carbon dioxide
SpO_2_: Oxygen saturation
